# Exploring the ^31^P chemical shift behavior of high-energy phosphates at 7 T in patients with glioma

**DOI:** 10.3389/fnins.2025.1638322

**Published:** 2025-10-31

**Authors:** Vanessa L. Franke, Florian Kroh, Bela Seng, Justyna Platek, Nina Weckesser, Heinz-Peter Schlemmer, Mark E. Ladd, Peter Bachert, Daniel Paech, Andreas Korzowski

**Affiliations:** ^1^Division of Medical Physics in Radiology, German Cancer Research Center (DKFZ), Heidelberg, Germany; ^2^Department of Radiology, Brigham and Women’s Hospital, Harvard Medical School, Boston, MA, United States; ^3^Faculty of Physics and Astronomy, Heidelberg University, Heidelberg, Germany; ^4^International Max Planck Research School for Quantum Dynamics in Physics, Chemistry, and Biology (IMPRS-QD), Max Planck Institute for Nuclear Physics (MPIK), Heidelberg, Germany; ^5^Division of Radiology, German Cancer Research Center (DKFZ), Heidelberg, Germany; ^6^Faculty of Medicine, Heidelberg University, Heidelberg, Germany

**Keywords:** phosphorus magnetic resonance spectroscopic imaging (^31^P MRSI), ^31^P chemical shifts, 7 tesla, glioma, IDH mutation, adenosine 5′-triphosphate, inorganic phosphate

## Abstract

**Introduction:**

The characterization of tumor microenvironment in vivo can be supported by ^31^P MRSI, a non-invasive technique that enables the determination of intracellular pH and magnesium ion concentration, among other parameters. However, it remains unclear from recent studies whether imaging biomarkers, like the intracellular pH value (as determined conventionally via the chemical shift separation between inorganic phosphate (P_i_) and phosphocreatine), are correlated with different glioma subtypes. Therefore, this study aimed to explore the behavior of multiple chemical shifts, specifically those of P_i_ and adenosine triphosphate (ATP), to approach a more detailed characterization of glioma tissues.

**Methods:**

A retrospective analysis on ^31^P MRSI datasets from 11 patients with newly diagnosed glioma acquired at 7 T prior to any treatment was conducted. Mean values of the quantified chemical shifts of P_i_, γ-, α- and β-ATP across different regions-of-interest were determined for each patient separately. The mean chemical shifts were compared for different tumor sub-compartments and for different IDH mutation status.

**Results:**

In high-grade gliomas, significant differences in chemical shifts were observed between tumor and healthy tissue. In low-grade glioma, smaller differences were found for the chemical shifts of P_i_, γ- and α-ATP than in the high-grade glioma. The latter pattern was not observed for β-ATP resonances, where the mean chemical shift across the tumor was comparably high between low-grade and high-grade glioma. In patients with IDH-wildtype, slightly stronger shifts of P_i_ and γ-ATP peaks were observed than for patients with IDH-mutant. No differences between IDH-wildtype and IDH-mutant were observed for the chemical shifts of α- and β-ATP.

**Discussion:**

These findings suggest a potential benefit of a joint evaluation of P_i_ and ATP chemical shifts for possible discrimination of different glioma subtypes. Using the complementary information of multiple ^31^P chemical shifts could improve the characterization of tumor tissue and provide new insights beyond current knowledge.

## 1 Introduction

With the progress toward more personalized approaches in precision medicine, the importance of imaging biomarkers that help to better characterize different diseases non-invasively is steadily increasing. In particular in cancer research, the search for imaging biomarkers plays an important role not only for the diagnosis and classification of tumors, but also for monitoring treatment response to novel targeted therapies (Platt et al., 2021; Shaffer et al., 2022). One promising technique for non-invasive imaging of such biomarkers is phosphorus magnetic resonance spectroscopic imaging (^31^P MRSI). With ^31^P MRSI the resonance signals of phosphorus-containing metabolites are investigated that are involved in energy metabolism [e.g., adenosine-triphosphate (ATP)] and membrane phospholipid turnover. Specifically, parameters such as the pH value or the magnesium ion concentration [Mg^2+^] can be indirectly assessed based on the quantifiable dependencies of the ^31^P resonance frequencies on the chemical environment. The potential of ^31^P MRSI to provide several interesting imaging biomarkers has already been demonstrated in various studies (Korzowski et al., 2021; Rijpma et al., 2018; Wenger et al., 2017).

In previous investigations (Paech et al., 2024) a positive correlation of the intracellular pH value (pH_i_) (as determined via the frequency difference between the resonances of inorganic phosphate (P_i_) and phosphocreatine (PCr) at 7T) with the proliferation marker Ki-67 has been reported. The Ki-67 marker is assumed to be linked to the aggressiveness of glioma. However, in this previous study (Paech et al., 2024), as well as in other studies (Galijašević et al., 2021), the biomarker pH_i_ lacked the ability to differentiate the tumor grade or the mutation status. This poses the question, whether the imaging biomarker pH is unrelated to specific tumor subtypes, or whether other effects are masking a correlation between pH and the tumor subtype. In this context, the chemical shift response of ATP, which can be used to estimate the magnesium ion concentration (Golding and Golding, 1995; Gupta et al., 1978) - but is also affected by pH - might provide additional insights. Up to now, the responses of the individual ATP resonances remain underexplored.

The ability of ^31^P MRS(I) to characterize simultaneously multiple aspects of the cellular microenvironment non-invasively, by means of pH and ionic composition (Franke et al., 2024a), might provide a new set of clinically interesting biomarkers for tumor tissues in a single scan. The stronger chemical shift dispersion at higher field strengths enables to analyze potentially finer differences in microenvironments, making the investigation of ^31^P chemical shift variation in pathologies at 7T of relevance. Thus, the extension beyond conventional ^31^P-based metrics – i.e., the joint analysis of P_i_ and ATP chemical shifts at ultra-high fields – might provide new discriminating features for different tumor subtypes.

To explore the potential of such an approach, a retrospective analysis of the chemical shifts of P_i_ and ATP was performed in ^31^P MRSI datasets of patients with newly diagnosed glioma obtained at 7T. The quantified chemical shifts of P_i_ and ATP were compared (i) for different tumor sub-compartments, and (ii) between patients with different mutation status of isocitrate dehydrogenase (IDH). Using a new version of a recently proposed look-up algorithm for the determination of pH and magnesium ion content under different conditions (Franke et al., 2024b; Seng et al., 2024), an attempt was taken to link the observed chemical shift differences to potential differences in the underlying biochemical parameters.

## 2 Materials and methods

### 2.1 ^31^P MRSI datasets used for the analysis of chemical shift behavior

For the investigation of chemical shifts in tumor and healthy tissue, ^31^P MRSI datasets from a cohort of 11 patients with newly diagnosed glioma were analyzed retrospectively. All datasets were originally acquired for a study recently published in Korzowski et al. (2021) and Paech et al. (2024). All examinations of this study were approved by the local ethics committee of the Medical Faculty of Heidelberg University, and written informed consent was received from all subjects. The ^31^P MRSI measurements were performed prior to any treatment on a 7T whole-body MR scanner (Magnetom 7T, Siemens Healthineers, Erlangen, Germany) using a double-resonant ^31^P-^1^H phased-array head coil with 32 ^31^P receiver elements (Rapid Biomedical, Rimpar, Germany). The ^31^P MRSI datasets were acquired with a 3D FID-MRSI sequence with the following parameters: TR = 250 ms, α = 20°, Δf = 5000 Hz, vectorsize = 1024, Hamming-weighted k-space sampling and transient ^31^P-^1^H nuclear Overhauser effect (NOE) enhancement via adiabatic water proton inversion. All datasets were acquired with isotropic spatial resolution, but partly with different matrix sizes, field of views, and number of *k*-space averages: 9 out of 11 datasets were acquired with a nominal resolution of (1.25 cm)^3^and 18 averages in the *k*-space center. To shorten the total protocol duration in two cases, one dataset was acquired with a nominal resolution of (1.3 cm)^3^ and 15 averages in the *k*-space center, and one dataset was acquired with a spatial resolution of (1.4 cm)^3^ and 24 averages in the *k*-space center. The total measurement time for the ^31^P MRSI scans was between 28 and 52 min. For B_0_ homogenization across the entire brain, 2*^nd^* order vendor-provided 3D gradient echo field mapping using the unsuppressed water resonance was conducted. Depending on the resulting field map, shimming was repeated iteratively a second or third time after readjusting the ^1^H reference frequency. Additionally to the ^31^P MRSI acquisition, 3D T1-weighted ^1^H images were acquired using an MPRAGE sequence (nominal spatial resolution = (1.4 mm)^3^, TR = 3.4 s, TI = 1.2 s, TE = 1.46 ms, α = 6°) (Korzowski et al., 2021) that were coregistered with the clinical images acquired at 3 T. Further details can be found in reference (Paech et al., 2024).

The ^31^P MRSI datasets were processed and quantified using MATLAB 2020a (The Mathworks, Natick, Massachusetts, USA). Before quantification, all datasets were processed by low-rank filtering, one-fold spatial zero-filling and application of a 15-Hz Gaussian filter in the time domain. Quantification of the ^31^P MRSI datasets was performed by using a home-built implementation of the AMARES algorithm (Vanhamme et al., 1997) for MATLAB (Korzowski et al., 2020). The fit model was the same for all datasets and included 13 resonance signals: PCr, γ-, α- and β-ATP, P_i_, extracellular P_i_ (eP_i_), phosphoethanolamine (PE), phosphocholine (PC), glycerophosphocholine (GPC), glycerophosphoethanolamine (GPE), 2,3-diphosphoglycerate (DPG), mobile phospholipids (MPL), nicotinamide adenine dinucleotide [NAD(H)], and uridine diphosphoglucose (UDPG). For the full description of the processing and quantification steps, the reader is referred to Korzowski et al. (2021).

### 2.2 Comparison of chemical shifts for different histopathology

Before comparing the chemical shifts, all quantified voxels underwent an automated quality assurance based on the calculated Cramer-Rao lower bounds (CRLBs). Only voxels for which the CRLBs of the quantified amplitudes of P_i_, γ-, α- and β-ATP were below 35% were included.

Mean values of the quantified chemical shifts of P_i_, γ-, α- and β-ATP across different regions-of-interest (ROIs) were determined for each patient separately. The comparison of chemical shifts was carried out for two different classifications.

First, the calculated mean chemical shifts were grouped into different tumor sub-compartments:

- gadolinium contrast enhancement (CE) for high-grade glioma (HGG; World Health Organization (WHO) grade III and IV, *n* = 8)
- edema (EDM) for high-grade glioma (HGG; World Health Organization (WHO) grade III and IV, *n* = 8)
- non-enhancing T_2_ hyper intensity (NCE) for low-grade glioma (LGG; WHO grade II, *n* = 2)
- normal-appearing white matter (WM) from all patients (including the patient with pleomorphic glioma; *n* = 11) as control

Second, it was investigated whether the ^31^P chemical shifts differ for patients with different IDH mutation status. For this purpose, the mean chemical shifts across the ROIs covering the whole tumor volume (WHT) were grouped into patients with IDH-mutant (*n* = 3), and IDH-wildtype (*n* = 7).

Note that the patient with pleomorphic glioma (patient #11 in Supplementary Table 1) was not included in the first analyses due the indefinite assignment to a specific tumor grade. In the second analysis (comparison of the different IDH mutation status), the mean values of the chemical shifts of the patient with pleomorphic glioma are shown separately as a green diamond (cf. Figure 4).

The tumor-grade classification was done based on the 2016 WHO classification of tumors (Louis et al., 2016) in accordance with the classification in our previous publication (Paech et al., 2024). Note, that the classification would not change using the 2021 classification. The volumetric segmentation of the listed ROIs was performed by two radiologists in consensus. Further information on the volumetric segmentation can also be found in reference (Paech et al., 2024). A more detailed description of the patient’s characteristics is given in Supplementary Table 1.

As an additional control measure, we also included mean chemical shift values from healthy white matter obtained from ^31^P MRSI datasets acquired in 6 healthy volunteers in the analysis. The ^31^P MRSI datasets from healthy controls were acquired with the long measurement protocol used for the majority of the patients (cf. section “2.1 ^31^P MRSI datasets used for the analysis of chemical shift behavior”). The segmentation of white matter in these datasets from healthy controls was performed using a home-built, automatic segmentation tool (Platek et al., 2023), which combines an automated segmentation for gray and white matter based on T1 and T2 weighted images (Ashburner and Friston, 2005) and a brain atlas provided by the SPM extension “automated anatomical labeling atlas” (Rolls et al., 2020). Note, that the age range in the cohort of healthy controls was [23–35] years, whereas for the patient cohort the age ranged between [23–80] years.

### 2.3 Calculation of pH value and magnesium ion content

In addition to the comparison of the chemical shifts, estimated values for pH and the magnesium ion content (Mg) were determined in the respective ROIs for each patient separately. This was done by means of a look-up algorithm as recently proposed (Franke et al., 2024a), but with novel look-up elements (Seng et al., 2024). The algorithm that includes the novel look-up table elements is provided in Franke et al. (2024b) as version 2.0. For the determination of pH and Mg values, the mean chemical shifts of P_i_, γ- and β-ATP relative to the chemical shift of α-ATP were used as input for the look-up algorithm. The resulting output values for pH, Mg and the parameter Ion (being a measure for the ionic strength) were also compared for the same classifications as described in the previous section (i.e., first different tumor sub-compartments, and second different IDH mutation status). Note, that the parameter Ion is a relative measure for the ionic strength in general, and further details can be found in references (Franke et al., 2024a; Seng et al., 2024).

### 2.4 Statistical analyses

The limited number of samples included in this study prevents to draw definitive conclusions about significant differences in chemical shifts for different tumor tissue types, particularly with respect to the LGG cases (*n* = 2). However, for comparisons with sufficient statistical power (>0.8), differences identified as significant according to the respective statistical test (i.e., paired *t*-test, independent *t*-test, or Mann-Whitney U test) are indicated in the text and figures, where appropriate.

A detailed description of the statistical methods applied, along with a comprehensive overview of all statistical parameters calculated across the study cohort, is provided in Supplementary Material 5.

## 3 Results

**FIGURE 1:**
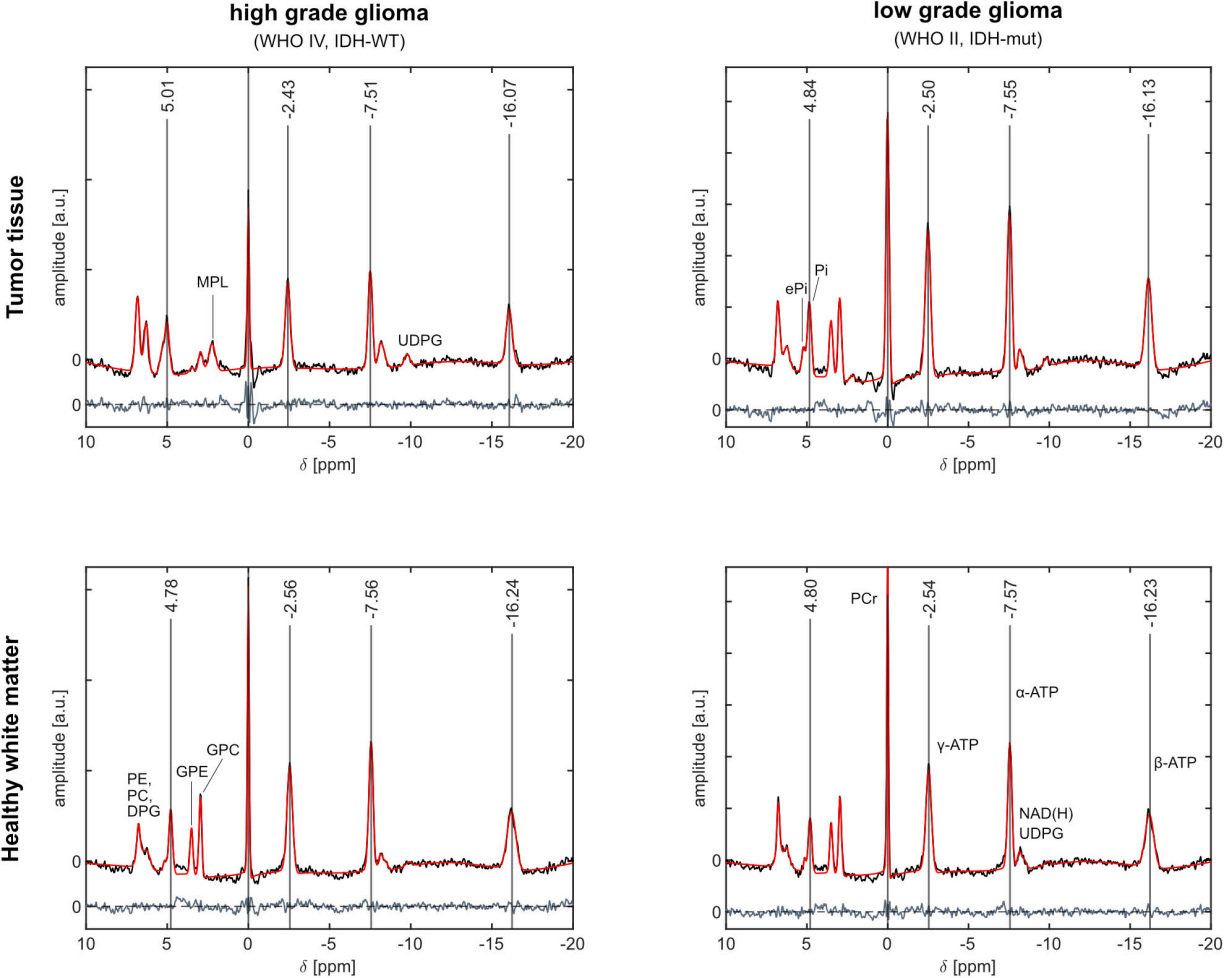
Representative ^31^P MR spectra from a patient with high-grade glioma (WHO grade IV, IDH-WT; left column) and from a patient with low-grade glioma (WHO grade II, IDH-mut; right column). The fitted signals (red) are overlaid with the measured signals (black) (all corrected for B_0_ field shifts). Additionally, the fitting residuum is shown below the spectra (gray). The quantified chemical shifts of P_i_ and ATP are marked as vertical lines. In the spectra from tumor tissue, the resonance peaks of P_i_, γ-, α- and β-ATP are shifted downfield compared to spectra from healthy white matter. The shifts in tumor tissue are larger for the high-grade glioma compared to the low-grade glioma. The chemical shifts obtained in healthy tissue show only minor differences between both cases (max. difference 0.02 ppm). The locations of the selected voxels in tumor tissue are shown in Figures 2, 3, respectively.

**FIGURE 2:**
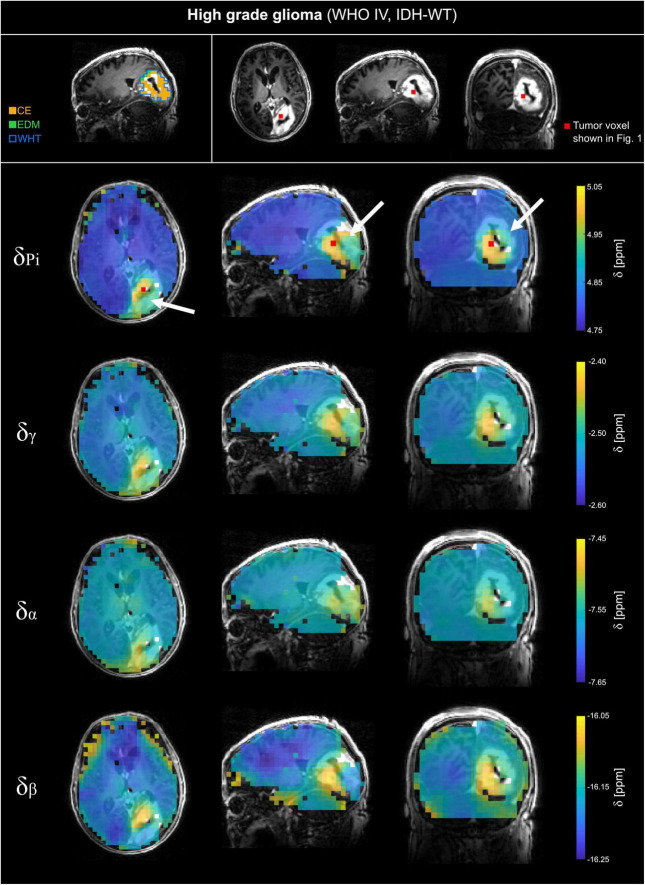
Volumetric maps of the quantified chemical shifts of inorganic phosphate (P_i_) and ATP (γ-, α- and β-ATP) relative to phosphocreatine of a representative patient with high-grade glioma (WHO grade IV, IDH-WT; HGG). The ^31^P MRSI datasets were acquired at B_0_ = 7T with an isotropic, nominal spatial resolution of (12.5 mm)^3^ and spatially zero-filled with a factor of 2 before quantification. The position of the selected tumor voxel shown in Figure 1 is indicated as red box on the slices of the anatomical image. Voxels that were outside the tissue mask or have been excluded from further analysis based on the automated quality assurance were made transparent. On the top left, the regions of interests (ROI) used for further analysis are shown for a representative slice. For patients with HGG, the ROIs covering the whole tumor volume (WHT), the gadolinium contrast enhanced area (CE), and the edema (EDM) were analyzed. The position of the tumor is highlighted with a white arrow.

**FIGURE 3:**
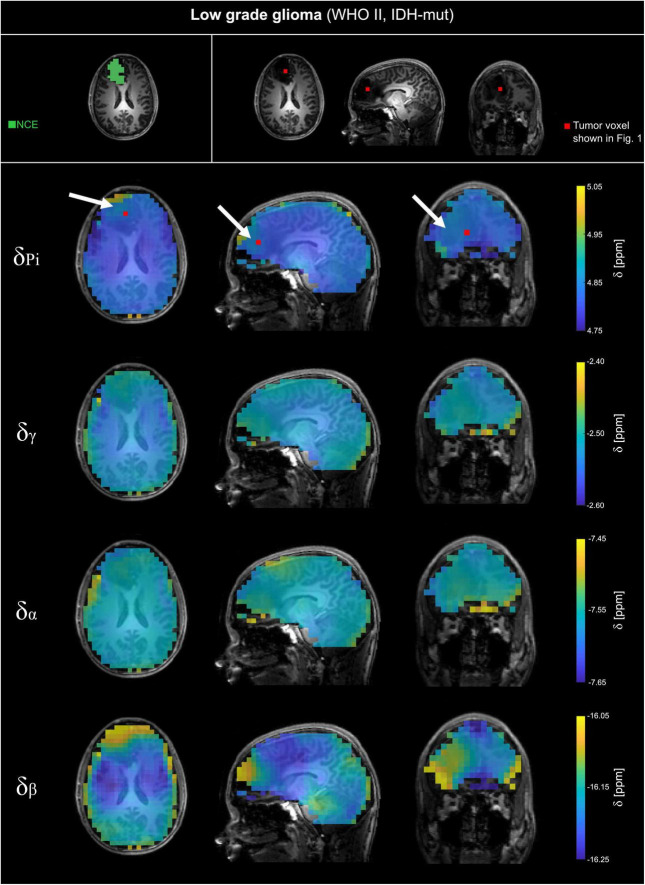
Volumetric maps of the quantified chemical shifts of inorganic phosphate (P_i_) and ATP (γ-, α- and β-ATP) relative to phosphocreatine of a representative patient with low-grade glioma (WHO grade II, IDH-mut; LGG). The ^31^P MRSI datasets were acquired at B_0_ = 7T with an isotropic, nominal spatial resolution of (12.5 mm)^3^ and spatially zero-filled with a factor of 2 before quantification. The position of the tumor voxel shown in Figure 1 is indicated as red box on the slices of the anatomical image. On the top left, the regions of interests (ROI) used for further analysis are shown for a representative slice. For patients with LGG, the ROIs covering non-enhancing T2 hyper intensity (NCE) were analyzed. The position of the tumor is highlighted with a white arrow.

The analyzed ^31^P MRSI datasets from patients with glioma reveal differences in chemical shifts for different tissues, which are visible when comparing the acquired spectra (cf. Figure 1). The quantified chemical shifts of P_i_ and ATP (measured relative to PCr) show differences between tumor and healthy tissue (cf. Figures 1–3). The resonances of P_i_, γ- and β-ATP obtained in tumor tissue are shifted downfield compared to those from healthy tissue. The α-ATP chemical shift shows minor differences between tumor and healthy tissue (maximal difference ≈ 0.05 ppm). For the chemical shifts of P_i_, γ- and α-ATP, the difference between tumor and healthy tissue is smaller for the patient with low-grade glioma (LGG) than for the patient with high-grade glioma (HGG). Interestingly, this is not valid for the chemical shift of β-ATP, where the difference between tumor and healthy tissue of the patient with LGG (Figure 3), is comparable to the chemical shift difference for the patient with HGG (Figure 2). The difference in δ_β_ between tumor and healthy tissue is up to 0.15 ppm for both patients. These qualitatively described observations are also confirmed in the following group analyses.

**FIGURE 4:**
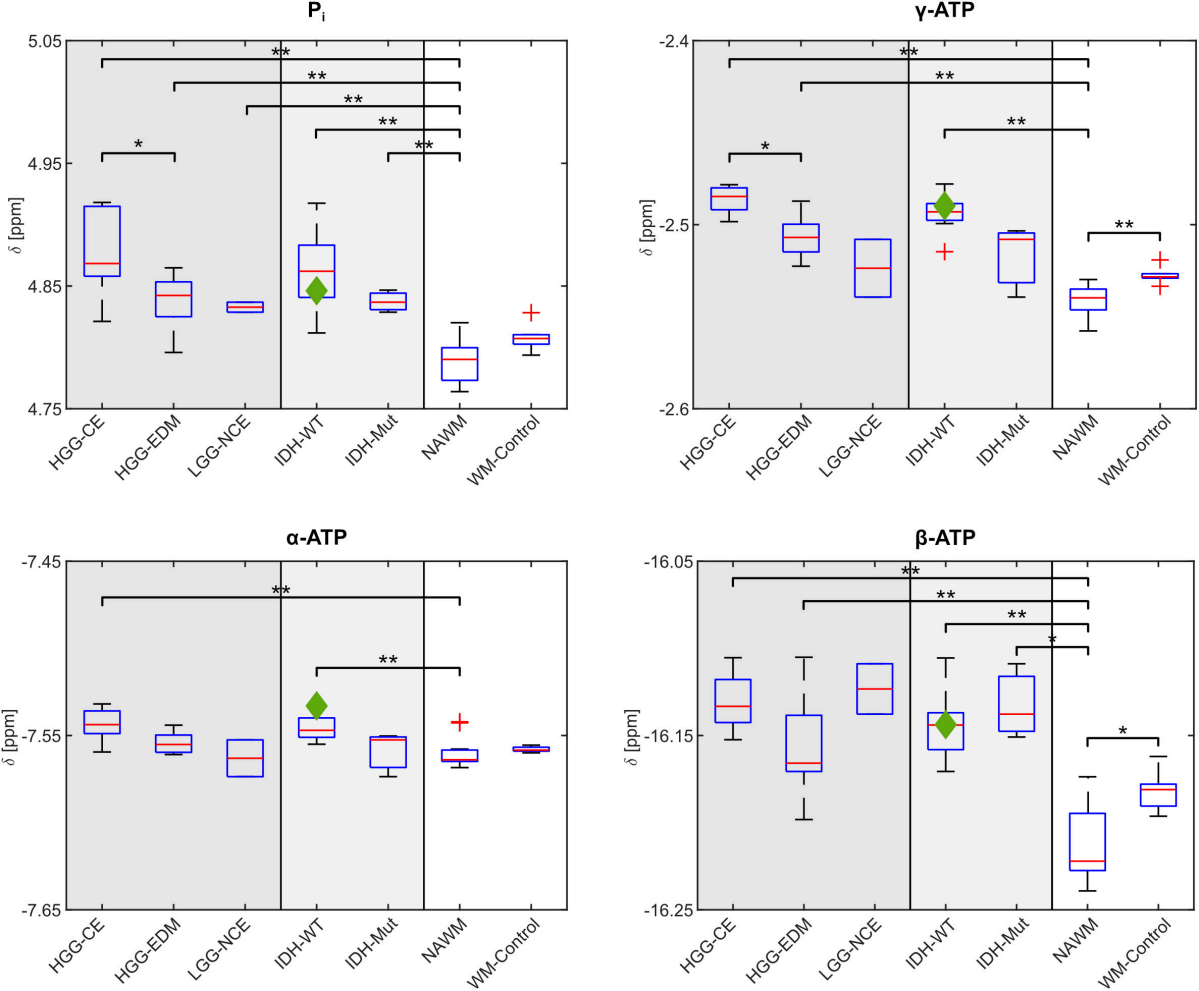
Comparison of the mean chemical shifts of P_i_ and ATP averaged across different regions-of-interest (ROIs) and different groups of patients. In the left sections, different tumor sub-compartments are compared: For patients with high-grade glioma (HGG, *n* = 8), the mean chemical shifts across the contrast enhancing areas (HGG-CE) and the edema (HGG-EDM) are shown, and for the patients with low-grade glioma (LGG, *n* = 2) the mean chemical shifts across the respective non-contrast enhanced areas (LGG-NCE). In the central sections, the mean chemical shifts across the respective whole tumor volume are compared between patients with different IDH-mutation status: IDH-wildtype (WT) (*n* = 7) and IDH-mutant (mut) (*n* = 3). The data point belonging to the dataset from the patient with pleomorphic glioma is marked as green diamond. In the right sections of each plot, the mean chemical shifts across the normal-appearing white matter ROI (NAWM) from all 11 patients are shown (here also the patient with pleomorphic glioma is included), as well as from a control group of healthy volunteers (WM-Control; *n* = 6). The red bar of the boxplots depicts the median across the respective groups of patients. Significant differences in mean chemical shifts with sufficient statistical power (>0.8; details in Supplementary Material 5) are indicated with *p < 0.05 and **p < 0.001.

Compared to NAWM, most of the investigated chemical shifts show significant differences in tumor tissue (cf. Figure 4). When compared to values from healthy white matter of the control group (WM-Control), the chemical shifts of γ- and β-ATP are significantly lower in the NAWM of tumor patients. Note, that the control group is not age-matched to the patient cohort.

The comparison of the mean chemical shifts across the different tumor sub-compartments (Figure 4, left sections) shows particular patterns in the chemical shifts. For patients with HGG, δ_Pi_, δ_γ_, δ_α_ and δ_β_ are generally higher in the CE-ROI than in the EDM-ROI, with a significant difference for δ_Pi_ (*p* = 0.014) and δ_γ_ (*p* = 0.004). For patients with LGG, δ_Pi_, δ_γ_ and δ_α_ are lower in the non-contrast enhanced ROI (LGG-NCE) compared to both HGG-GCE and HGG-EDM, but still higher than for healthy tissue. In contrast to that, for δ_β_, the shifts in the LGG-NCE ROI appear to be as high as the shifts in the HGG-CE ROI, supporting the observations from the representative datasets in Figures 1–3.

The comparison of the chemical shifts between groups of patients with different IDH-mutation status (Figure 4, central sections) shows slightly stronger shifts of P_i_, α- and γ-ATP for patients with IDH-WT than for patients with IDH-mut. For the chemical shift of β-ATP, no difference between IDH-WT and IDH-mut is observed.

**FIGURE 5:**
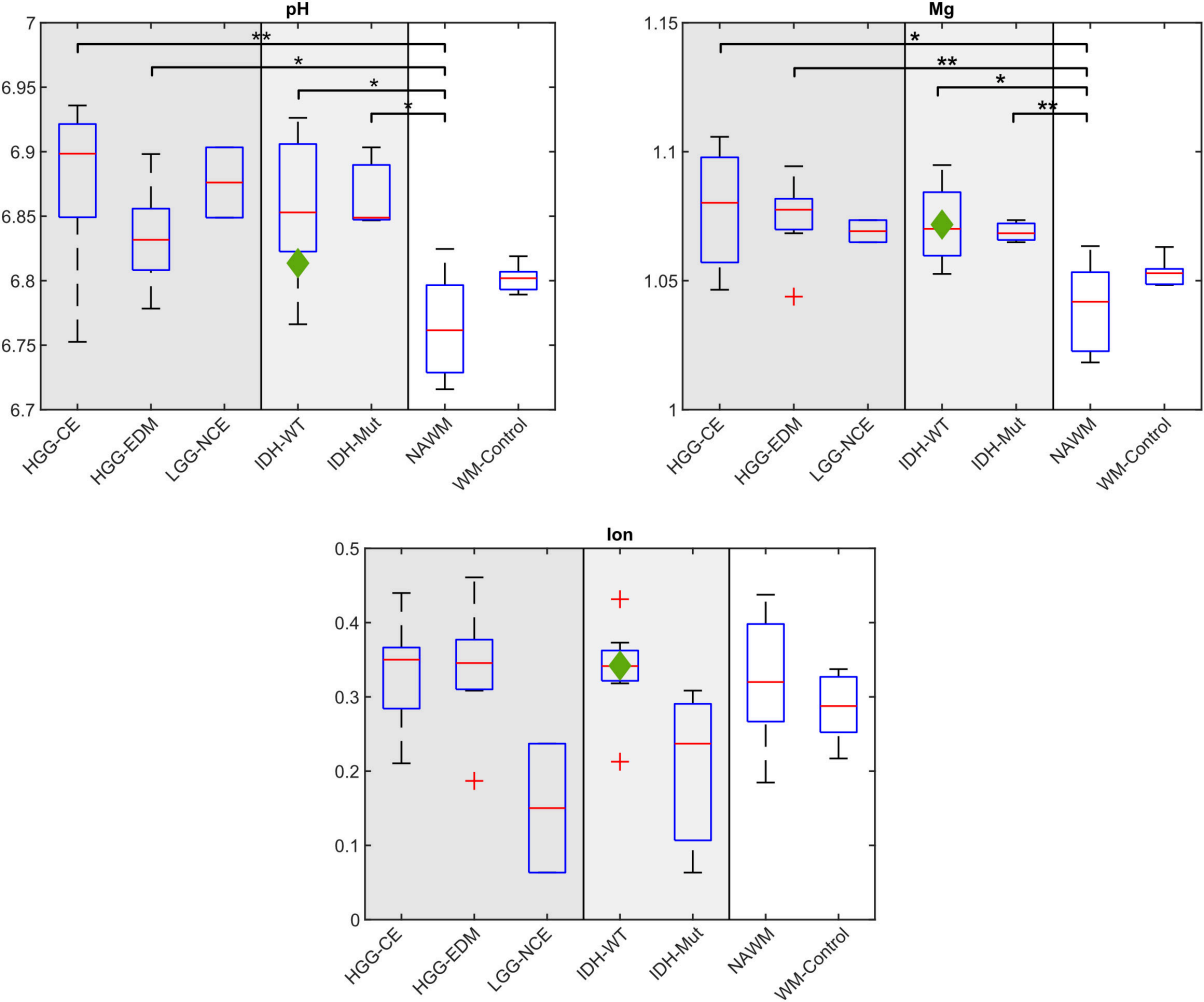
Comparison of the determined values for pH, magnesium ion content (Mg) and the parameter Ion for the different regions-of-interest (ROIs) and different groups of patients. In the left sections, different tumor sub-compartments are compared: For patients with high-grade glioma (HGG, *n* = 8), the values for (pH, Mg, Ion) determined from the mean chemical shifts in the contrast enhancing areas (HGG-CE) and the edema (HGG-EDM) are shown, and for patients with low-grade glioma (LGG, *n* = 2) from the non-contrast enhanced areas (LGG-NCE). In the central sections, the determined values (pH, Mg, Ion) from the respective whole tumor volume are compared between patients with different IDH-mutation status: IDH-wildtype (WT) (*n* = 7) and IDH-mutant (mut) (*n* = 3). The data point belonging to the dataset from the patient with pleomorphic glioma is marked as green diamond. In the right sections of each plot, the mean chemical shifts across the normal-appearing white matter ROI (NAWM) from all 11 patients are shown (here also the patient with pleomorphic glioma is included), as well as from a control group of healthy volunteers (WM-Control; *n* = 6). The red bar of the boxplots depicts the median across the respective groups of patients. Significant differences with sufficient statistical power (> 0.8, details in Supplementary Material 5) are indicated with *p < 0.05 and **p < 0.001.

The values for pH and the magnesium ion content obtained with the look-up algorithm are significantly higher in the tumor tissue compared to NAWM (except for the comparison LGG-NCE/NAWM, where the statistical power is insufficient) (cf. Figure 5). The parameter Ion appears to be around the same level in tumor and healthy tissue for patients with IDH-WT (cf. Figure 5). Compared to that, for patients with IDH-mut, the parameter Ion tends to lower values in the tumor compared to NAWM (cf. Figure 5).

When comparing the determined values for pH, Mg and Ion in the different tumor sub-compartments (Figure 5, left sections), no differences between the compartments can be observed for pH and Mg. However, the parameter Ion appears to be decreased in the LGG-NCE ROI compared to both the HGG-CE and HGG-EDM ROI.

## 4 Discussion

In this study, we explored the behavior of the ^31^P chemical shifts of P_i_ and ATP in glioma tissue, in order to identify potentials to differentiate between different glioma subtypes.

For high-grade glioma, we found significant chemical shift differences in the tumor region compared to healthy tissue (with moderate differences in edema). For low-grade glioma, smaller differences were detected than for the high-grade glioma for P_i_, γ- and α-ATP. Interestingly, this behavior was not observed for β-ATP resonances – here, the mean chemical shift across the tumor of the low-grade glioma appears to be as high as in the high-grade glioma. This deviation might contain additional information, as is discussed in the following.

### 4.1 Interpretation of the observed behavior in the investigated chemical shifts

It is known that ^31^P chemical shifts are influenced by changes in biochemical parameters, i.e., the pH value, the magnesium ion concentration, and the concentration of other ions (Franke et al., 2024a; Pettegrew et al., 1988). Overall, these parameters lead to a downfield shift when they are increased, but – very importantly – with varying extent.

The pH value has the strongest influence on the shifts of P_i_ and γ-ATP, followed by β- and α-ATP. From earlier studies investigating pH values using the conventional method based only on the P_i_-PCr shift difference, a gradual increase in intracellular pH values from healthy tissue over LGG/IDH-mut to HGG/IDH-WT tumors could be expected (Paech et al., 2024; Wenger et al., 2017). Hence, a gradually increasing downfield shift of P_i_ and ATP is expected, which was indeed observed in this study for P_i_, γ- and α-ATP, but not for β-ATP. Instead, in all patients (LGG and HGG) a similarly high δ_β_ was observed in tumor tissue compared to healthy tissue. It is important to note that all ATP chemical shifts are strongly influenced by the magnesium ion concentration, whereby δ_β_ shifts the most with increasing Mg, followed by δ_γ_ and δ_α_. The chemical shift of P_i_ is only slightly influenced by Mg. An alteration in free magnesium ion concentration in tumor tissue has been previously reported (Galijašević et al., 2021; Taylor et al., 1991). Thus, the observed “high” δ_β_ could be attributable to a higher free magnesium ion content in the tumor tissue of the patients in this study, which would then be unspecific to the glioma subtype. However, this assumption is partly contradicted by the observed (non-significant) chemical shift differences of γ- and α-ATP between the LGG and HGG groups (although the difference in γ-ATP shifts could possibly also be driven dominantly by a strong pH difference).

Taking into account the abovementioned effects of both pH and Mg with varying effect strengths, the observed behavior in chemical shifts of P_i_ and ATP are difficult to explain by these two parameters alone, rather pointing toward further influential factors. The influence of other ions on the ^31^P chemical shifts has been investigated less intensively, but it is known to be smaller than the influence of pH and Mg. Na^+^ and K^+^ have been reported to influence the shifts of γ- and β-ATP stronger than those of α-ATP and P_i_ (Mottet et al., 1994; Roberts et al., 1981). In this context, it is interesting to mention a study by Regnery et al. (2020) employing ^23^Na MRI at 7T in glioma patients, where differences in the total sodium concentration were observed between patients with high-grade and low-grade gliomas, as well as between patients with different IDH mutation status (higher total sodium in LGG/IDH-mut). Therefore, it seems reasonable to assume an alteration in ionic compositions also across our patient cohort, which might possibly explain the different chemical shift signatures of P_i_ and ATP in the different patient groups. For example, a decrease in ionic strength would result in a decrease in δ_γ_, compensating a potential increase due to an elevated pH. The latter could explain the unobserved elevated δ_γ_ shift in the LGG compared to the HGG, while maintaining similar β-ATP chemical shift.

These observations match with the results of the look-up algorithm attempting to provide potential biochemical explanations for the observed behavior: While the determined values for pH and Mg do not seem to differ strongly between the different groups, a difference between groups appears to exist for the parameter Ion (also differing from NAWM). Note that the implemented look-up algorithm provides the parameter Ion as a surrogate for ionic strength in general, not giving a specific concentration of one species. But as it is weighted by the K^+^ concentration [cf. reference (Franke et al., 2024a)], the observed decrease in the parameter Ion in the LGG/IDH-mut patients does not necessarily contradict the increased total Na^+^ content reported by Regnery et al. (2020) (alteration of Na^+^/K^+^ gradient). Based on these considerations, it seems reasonable to account for changes in all three biochemical parameters – pH, Mg, and Ion – simultaneously when attempting to discriminate and characterize different glioma subtypes by means of ^31^P MRSI. Hence, a joint analysis of multiple ^31^P chemical shifts is reasonable in future studies instead of analyzing the shifts individually (as in the conventional pH and Mg estimations).

### 4.2 Limitations and future outlook

Regarding the determined biochemical parameters, it is important to note that the estimated pH values determined by the look-up algorithm are systematically shifted by approximately −0.1 pH units compared to the pH values calculated using the modified Henderson-Hasselbalch equation which exploits the chemical shift difference between P_i_ and PCr only. This mismatch presumably results from a systematic difference between the prepared model solutions used for the development of the look-up algorithm and the *in vivo* conditions. This was thoroughly discussed in previous publications (Franke et al., 2024a; Seng et al., 2024). These systematic differences must be investigated in future studies. Nevertheless, this issue only influences the determined absolute values for the biochemical parameters, while the overall relative differences are preserved – which are the main focus of this study. Even if the absolute values might be systematically shifted, the relative ^31^P chemical shifts measured accurately from tumor tissue justify the biochemical considerations that were made in this work. Further, note that uncertainties in reported chemical shifts might arise from uncertainties in the AMARES quantification as determined via the Cramer-Rao lower bounds (CRLBs). Because the majority of CRLBs for the analyzed chemical shifts (in the order of 0.01 ppm or less; cf. Supplementary Material 2) are smaller than the spectral resolution of the acquisition (∼0.04 ppm) and smaller than the mean differences, this quantification uncertainty in chemical shift is rendered negligible in the context of this study.

We are aware of the small cohort size in this study comprising evaluated datasets of only 11 patients. However, even in this small group, for same cases, significant differences in the ^31^P chemical shifts for different tumor tissue types were observed, motivating further investigations with a larger cohort size in the future. With an approach as performed in this study, an extended characterization of the tumor microenvironment based on ^31^P MRSI could be tested, and a classification into different sub-cohorts might be possible. However, a significant reduction in measurement time of the ^31^P MRSI scans to about 15 min would be required to enable integration into large-scale clinical studies. In this regard, future work should especially focus on suitable acceleration methods for ^31^P MRSI without compromising the high spectral resolution needed for such a stratification as performed in this study. Besides repeating this study with a larger patient cohort in order to investigate whether the observed differences are significant, an acceleration of ^31^P MRSI also aids the comparison and cross-validation with other methods. In particular using X-nuclei MRI, e.g., with ^23^Na or ^39^K, in combination with ^31^P MRSI would enable to directly correlate measured ion concentrations *in vivo* to the measured ^31^P chemical shift signatures.

Based on the findings of this study, we hypothesize that the addition of complementary information provided by the quantified chemical shifts of P_i_ and ATP – yielding specific chemical shift signatures – could potentially enable an improved discrimination of different tumor tissue types. Relevant questions in this regard would be (I) which combination of chemical shifts is most indicative for a separation of tumor grades or mutation status. To this end, methods like SVM or k-means clustering analyses on multiple shift differences should be employed. Additionally, in case of superior performance of such newly defined metrics, one could test (II) whether chemical shift signatures corresponding to tumor tissue indicate pathological areas being not visible on conventional morphology. These two clinically-relevant questions, however, require also normative reference values for chemical shifts from healthy subjects across different age spans, once again highlighting the need for larger study cohorts.

## 5 Conclusion

This retrospective study of ^31^P MRSI datasets acquired at 7 T in 11 patients with glioma revealed a noteworthy behavior of chemical shifts across different tumor tissue types, in particular the comparable shift of β-ATP across different glioma subtypes. This hints toward an influence of varying ionic composition in tumor tissues being detectable via ^31^P MRSI at 7T, and demonstrating a potential benefit of a joint exploitation of P_i_ and ATP chemical shifts for possible discrimination of different tissue types. Using the complementary information of multiple ^31^P chemical shifts might improve the characterization of tumor tissue beyond current knowledge, e.g., by determining specific chemical shift signatures for different tumor types in the future.

## Data Availability

The data analyzed in this study is subject to the following licenses/restrictions: DGSVO. Requests to access these datasets should be directed to corresponding author.
