# Supplementary material for: Exploring the 31P chemical shift behavior of high-energy phosphates at 7 T in patients with glioma
Source: Front Neurosci. 2025 Oct 31;19:1638322. doi: 10.3389/fnins.2025.1638322 (PMC12615441; doi:10.3389/fnins.2025.1638322)
Supplement: Supplementary file 1 [file Data_Sheet_1.pdf]

Supplementary Material

Exploring the ^{31}P chemical shift behavior of high-energy phosphates at 7T in patients with glioma

Vanessa L. Franke[1*], Florian Kroh[2], Bela Seng[1,3], Justyna Platek[1,3,4], Nina Weckesser[5], Heinz-Peter Schlemmer[5,6], Mark E. Ladd[1,3,6], Peter Bachert[1,3], Daniel Paech[2,5], and Andreas Korzowski[1]

[1]Division of Medical Physics in Radiology, German Cancer Research Center (DKFZ), Heidelberg, Germany

[2]Department of Radiology, Brigham and Women's Hospital, Harvard Medical School, Boston, MA, United States

[3]Faculty of Physics and Astronomy, Heidelberg University, Heidelberg, Germany

[4]International Max Planck Research School for Quantum Dynamics in Physics, Chemistry, and Biology (IMPRS-QD), Max Planck Institute for Nuclear Physics (MPIK), Heidelberg, Germany

[5]Division of Radiology, German Cancer Research Center (DKFZ), Heidelberg, Germany

[6]Faculty of Medicine, Heidelberg University, Heidelberg, Germany

*** Correspondence:**

Vanessa L. Franke

v.franke@dkfz.de

S1 – Characteristics of study participants

The analysis of data in this study was retrospectively performed on a cohort of 13 patients with glioma, previously reported in (1). From this cohort two datasets were excluded due to inadequate data quality (i.e. severe line broadening due to B_0 inhomogeneities in direct proximity to the paranasal sinuses in one case, and insufficient quantification of the ATP resonances in the other case) in this analysis resulting in a total of n = 11 datasets for further analysis. Note that compared to the work in reference 1, an additional patient was excluded due to insufficient quantification of the ATP resonances being of high relevance for the investigations in this study. The characteristic of the study participants of the finally analyzed datasets are described in Table S1. Note that the patient with pleomorphic glioma (#11) was not included in the comparison of tumor grades because of its indefinite assignment to a specific grade. In the comparison of different IDH mutation status, the values of this patient were also not included, but depicted separately in the corresponding figure. The values from healthy white matter were included in the statistics.

Table S1: Characteristics of study participants retrospectively analyzed. *The dataset from the patient with pleomorphic glioma (#11) was not included in the comparison between high-grade glioma and low-grade glioma because of its unclear assignment to a specific tumor grade.

Pat. No.	Age (y)	Sex	WHO Grade[2]	Histo-pathology	IDH Status	Tumor Location	Largest Ø (mm)
1	71	M	IV	Glioblastoma	WT	Right temporal/parietal	29.46
2	57	M	IV	Glioblastoma	WT	Left temporal/occipital	39.55
3	68	M	IV	Glioblastoma	WT	Right temporal/parietal	64.85
4	78	F	IV	Glioblastoma	WT	Left temporal	47.99
5	36	F	III	Astrocytoma	MUT	Right frontal	46.31
6	67	F	IV	Glioblastoma	WT	Right temporal	45.32
7	80	F	IV	Glioblastoma	WT	Left temporal	40.72
8	75	M	IV	Glioblastoma	WT	Left occipital	61.05
9	34	M	II	Astrocytoma	MUT	Left temporal	47.09
10	23	M	II	Astrocytoma	MUT	Right frontal	48.91
11*	50	M	-	Pleomorphic glioma	WT	Bifrontal	87.17

Abbreviations: IDH – Isocitrate Dehydrogenase; MUT – mutated; WHO – World Health Organization; WT – wildtype

S2 – Further data quality measures

In Tables S2.1 – S2.5, the Cramér-Rao Lower bounds (CRLB) of the fitted chemical shifts are given in [ppm] for all metabolites of interest and for each patient separately. Mean, median and maximum values of the CRLBs across the entire brain tissue mask, the whole tumor ROI and the healthy white matter ROI are provided.

In Table S2.6, fitted linewidth of phosphocreatine (PCr) in [Hz] are provided for each patient dataset. Listed are the mean values and standard deviation across the brain tissue mask, the whole tumor ROI and the healthy white matter ROI.

Table S2.1: Cramér-Rao Lower Bounds (CRLB) of the fitted chemical shifts in [ppm] for phosphocreatine (PCr) in the brain tissue masks, the whole tumor ROI and the normal-appearing white matter ROI for each patient. Listed are the mean, median and maximum values of the CRLBs across the tissue mask.

Chemical shift CRLB [ppm], PCr									
**Pat. No.**	**Brain Tissue Mask**			**Whole Tumor ROI**			**White Matter ROI**		
	mean	median	max	mean	median	max	mean	median	max
**1**	0.0012	0.0009	0.0195	0.0006	0.0006	0.0008	0.0007	0.0007	0.0007
**2**	0.0014	0.0010	0.0160	0.0014	0.0010	0.0066	0.0009	0.0009	0.0009
**3**	0.0014	0.0010	0.0225	0.0019	0.0013	0.0176	0.0009	0.0009	0.0010
**4**	0.0009	0.0007	0.0256	0.0013	0.0010	0.0070	0.0005	0.0005	0.0005
**5**	0.0014	0.0010	0.0160	0.0014	0.0012	0.0060	0.0004	0.0004	0.0004
**6**	0.0015	0.0011	0.0136	0.0013	0.0009	0.0091	0.0010	0.0010	0.0011
**7**	0.0013	0.0010	0.0174	0.0008	0.0007	0.0035	0.0008	0.0008	0.0010
**8**	0.0011	0.0008	0.0160	0.0008	0.0007	0.0024	0.0007	0.0007	0.0008
**9**	0.0010	0.0007	0.0191	0.0023	0.0018	0.0076	0.0006	0.0006	0.0007
**10**	0.0011	0.0008	0.0142	0.0009	0.0009	0.0033	0.0005	0.0005	0.0005
**11**	0.0011	0.0008	0.0245	0.0014	0.0010	0.0123	0.0005	0.0005	0.0006

Table S2.2: Cramér-Rao Lower Bounds (CRLB) of the fitted chemical shifts in [ppm] for inorganic phosphate (P_i) in the brain tissue masks, the whole tumor ROI and the normal-appearing white matter ROI for each patient. Listed are the mean, median and maximum values of the CRLBs across the tissue mask.

Chemical shift CRLB [ppm], P_i									
**Pat. No.**	**Brain Tissue Mask**			**Whole Tumor ROI**			**White Matter ROI**		
	mean	median	max	mean	median	max	mean	median	max
**1**	0.0097	0.0060	0.1382	0.0052	0.0052	0.0080	0.0033	0.0033	0.0034
**2**	0.0090	0.0056	0.2480	0.0112	0.0084	0.0401	0.0047	0.0047	0.0053
**3**	0.0095	0.0058	0.1961	0.0166	0.0109	0.1057	0.0049	0.0047	0.0061
**4**	0.0068	0.0047	0.1001	0.0096	0.0081	0.0514	0.0032	0.0031	0.0038
**5**	0.0106	0.0073	0.1162	0.0114	0.0087	0.0688	0.0041	0.0036	0.0053

**6**	0.0112	0.0072	0.2489	0.0127	0.0062	0.2489	0.0056	0.0056	0.0069
**7**	0.0104	0.0061	0.2181	0.0137	0.0082	0.0787	0.0041	0.0037	0.0062
**8**	0.0094	0.0055	0.3048	0.0118	0.0096	0.0529	0.0048	0.0042	0.0072
**9**	0.0075	0.0044	0.1476	0.0191	0.0091	0.1476	0.0046	0.0044	0.0062
**10**	0.0076	0.0045	0.1241	0.0055	0.0035	0.0265	0.0031	0.0030	0.0035
**11**	0.0080	0.0052	0.1298	0.0142	0.0102	0.0940	0.0027	0.0027	0.0029

Table S2.3: Cramér-Rao Lower Bounds (CRLB) of the fitted chemical shifts in [ppm] for γ-ATP in the brain tissue masks, the whole tumor ROI and the normal-appearing white matter ROI for each patient. Listed are the mean, median and maximum values of the CRLBs across the tissue mask.

**Chemical shift CRLB [ppm], γ-ATP**									
**Pat. No.**	**Brain Tissue Mask**			**Whole Tumor ROI**			**White Matter ROI**		
	mean	median	max	mean	median	max	mean	median	max
**1**	0.0051	0.0042	0.0485	0.0028	0.0028	0.0035	0.0035	0.0036	0.0038
**2**	0.0048	0.0037	0.0684	0.0042	0.0037	0.0143	0.0037	0.0037	0.0038
**3**	0.0042	0.0033	0.0420	0.0043	0.0034	0.0356	0.0031	0.0031	0.0032
**4**	0.0031	0.0026	0.0198	0.0031	0.0028	0.0062	0.0020	0.0021	0.0021
**5**	0.0041	0.0033	0.0314	0.0034	0.0031	0.0092	0.0022	0.0022	0.0022
**6**	0.0043	0.0034	0.0424	0.0030	0.0027	0.0122	0.0034	0.0035	0.0036
**7**	0.0041	0.0033	0.0399	0.0031	0.0028	0.0096	0.0025	0.0025	0.0027
**8**	0.0038	0.0030	0.0340	0.0025	0.0025	0.0039	0.0027	0.0027	0.0029
**9**	0.0033	0.0027	0.0281	0.0061	0.0046	0.0223	0.0026	0.0025	0.0034
**10**	0.0031	0.0026	0.0490	0.0023	0.0020	0.0125	0.0023	0.0023	0.0025
**11**	0.0036	0.0030	0.0317	0.0031	0.0027	0.0122	0.0027	0.0027	0.0030

Table S2.4: Cramér-Rao Lower Bounds (CRLB) of the fitted chemical shifts in [ppm] for α-ATP in the brain tissue masks, the whole tumor ROI and the normal-appearing white matter ROI for each patient. Listed are the mean, median and maximum values of the CRLBs across the tissue mask.

**Chemical shift CRLB [ppm], α-ATP**									
**Pat. No.**	**Brain Tissue Mask**			**Whole Tumor ROI**			**White Matter ROI**		
	mean	median	max	mean	median	max	mean	median	max
**1**	0.0044	0.0034	0.0759	0.0022	0.0022	0.0026	0.0027	0.0028	0.0029
**2**	0.0043	0.0028	0.1136	0.0035	0.0028	0.0183	0.0026	0.0026	0.0027
**3**	0.0039	0.0027	0.0766	0.0039	0.0028	0.0464	0.0022	0.0022	0.0024
**4**	0.0026	0.0020	0.0704	0.0026	0.0024	0.0073	0.0014	0.0014	0.0015
**5**	0.0040	0.0026	0.0895	0.0029	0.0027	0.0083	0.0015	0.0015	0.0015
**6**	0.0044	0.0028	0.0722	0.0027	0.0022	0.0111	0.0025	0.0026	0.0027
**7**	0.0036	0.0027	0.1175	0.0026	0.0022	0.0138	0.0018	0.0018	0.0019
**8**	0.0032	0.0023	0.0766	0.0021	0.0020	0.0037	0.0020	0.0020	0.0021

9	0.0029	0.0021	0.0591	0.0068	0.0046	0.0246	0.0018	0.0017	0.0029
10	0.0029	0.0020	0.0488	0.0020	0.0017	0.0123	0.0016	0.0016	0.0018
11	0.0033	0.0024	0.1147	0.0033	0.0022	0.0430	0.0018	0.0018	0.0019

Table S2.2: Cramér-Rao Lower Bounds (CRLB) of the fitted chemical shifts in [ppm] for β-ATP in the brain tissue masks, the whole tumor ROI and the normal-appearing white matter ROI for each patient. Listed are the mean, median and maximum values of the CRLBs across the tissue mask.

Chemical shift CRLB [ppm], β-ATP									
Pat. No.	Brain Tissue Mask			Whole Tumor ROI			White Matter ROI		
	mean	median	max	mean	median	max	mean	median	max
1	0.0114	0.0096	0.0826	0.0060	0.0060	0.0072	0.0085	0.0085	0.0090
2	0.0085	0.0069	0.0943	0.0074	0.0067	0.0218	0.0069	0.0069	0.0070
3	0.0076	0.0063	0.0626	0.0069	0.0060	0.0436	0.0059	0.0060	0.0062
4	0.0056	0.0049	0.0327	0.0052	0.0050	0.0115	0.0041	0.0041	0.0042
5	0.0071	0.0061	0.0500	0.0055	0.0053	0.0126	0.0048	0.0047	0.0048
6	0.0079	0.0063	0.0569	0.0053	0.0050	0.0136	0.0062	0.0062	0.0067
7	0.0071	0.0059	0.0552	0.0047	0.0044	0.0118	0.0047	0.0048	0.0051
8	0.0069	0.0056	0.0488	0.0042	0.0041	0.0058	0.0055	0.0055	0.0056
9	0.0062	0.0053	0.0735	0.0101	0.0089	0.0239	0.0050	0.0049	0.0053
10	0.0054	0.0048	0.0785	0.0034	0.0032	0.0144	0.0051	0.0052	0.0055
11	0.0063	0.0056	0.0617	0.0048	0.0044	0.0146	0.0057	0.0056	0.0063

Table S2.6: Fitted linewidth of phosphocreatine (PCr) in [Hz] for each patient separately. Listed are the mean and standard deviation across the brain tissue mask, the whole tumor ROI and the healthy white matter ROI.

PCr linewidths [Hz]						
Pat. no.	Brain Mask		Whole Tumor ROI		White Matter ROI	
	mean	std	mean	std	mean	std
1	11.9	4.9	10.2	1.4	8.6	0.3
2	13.8	6.5	14.0	6.4	9.7	0.3
3	13.8	5.2	15.6	6.7	12.3	0.6
4	12.0	4.3	12.2	3.7	9.2	0.5
5	13.0	6.3	12.2	3.8	7.6	0.2
6	15.7	5.9	15.0	5.8	12.4	0.3
7	14.0	5.5	12.0	3.3	12.6	1.0
8	12.3	5.4	11.3	3.9	9.9	0.7
9	12.8	5.0	17.6	6.0	9.5	0.4
10	14.0	5.5	15.8	4.0	8.6	0.3
11	13.1	5.7	14.8	7.0	9.2	0.4

S3 – Minimum Reporting Standards for in vivo MRS (MRSinMRS)

Table S3: Minimum Reporting Standards following Lin et al.(2)

**1. Hardware**	
a. Field strength [T]	7 T
b. Manufacturer	Siemens Healthineers, Erlangen, Germany
c. Model	Magnetom 7 T (VB17)
d. RF coils	Double-resonant ^{31}P-^{1}H phased-array head coil with 32 ^{31}P receiver elements (Rapid Biomedical, Rimpar, Germany)
**2. Acquisition**	
a. Pulse sequence	3D FID-MRSI with Hamming-weighted k-space acquisition
b. VOI location	Entire brain
c. Nominal VOI size [mm³]	N/A (whole-volume acquisition)
d. Repetition time (TR)	250 ms
e. Acquisitions	18 averages (in k-space center)
f. Additional sequence parameter	Bandwidth = 5 kHz, 1024 complex points after removing oversampling, flip angle = 20°
i. TM	N/A
ii. FOV / matrix size (x, y, z)	(250 x 300 x 250) mm³<br>(20 x 24 x 16)
iii. ^{1}H NOE	Yes (hyperbolic secant shaped inversion pulse, $B_{1,mean}$ ≈ 6 μT, T_{Pulse} = 6 ms, T_I = 10 ms)
g. Water suppression method	N/A

h. Shimming Method	2nd order vendor-provided 3D gradient echo fieldmap using the unsuppressed water resonance
i. Triggering/motion correction	N/A
**3. Data analysis methods and outputs**	
a. Analysis software	ustom-built AMARES(3) algorithm in MATLAB R2020a (The Mathworks, Natick, USA)
b. Processing steps deviated from product analysis software	Reconstruction, postprocessing and quantification was performed with custom-built scripts in MATLAB R2020a. For further details, see main manuscript section 2.1.
c. Output measure	Chemical shifts relative to PCr in [ppm], metabolite amplitudes in [a.u.], linewidths in [Hz]
d. Quantification references and assumptions	N/A
**5. Data quality**	
a. Reported variables	• The mean linewidth of PCr<br>• Relative CRLBs for amplitude and absolute CRLBs for the chemical shifts of P_i and ATP<br><br>(see Supplementary Material S2)
b. Data exclusion criteria	Relative CRLBs of the fitted amplitudes of P_i and ATP > 35%
c. Quality measures of postprocessing model fitting	See point 5a
d. Sample spectrum	Figure 1

S4 – Number of excluded voxels per patient

Tables S4.1 and S4.2 provide a detailed overview of how many voxels were excluded based on the CRLBs of the fitted amplitudes of P_i and ATP for each patient individually. The majority of excluded voxels were located at the edges of the manually drawn brain masks, mainly near the nasal cavities. In the healthy white matter ROIs, no voxels were excluded (cf. Table S4.1, 4th column).

In order to prevent any bias due to the exclusion of voxels solely on the relative CRLBs of the fitted amplitudes, the chemical shift analysis was conducted in the same way but without exclusion of voxels based on the CRLBs. Figure S4 shows the comparison of mean chemical shifts of P_i and ATP across the different ROIs, analogously to Figure 4 in the main manuscript. When comparing Figure 4 from the main manuscript with supplementary Figure S4, no major differences are visible. Due to the point-spread function (larger effective voxel size), the spectral signatures are most likely conserved in neighboring voxels resulting in no bias of the results with or without exclusion of voxels based on the amplitude CRLBs.

Table S4.1: Number of excluded voxels for the regions-of-interest (ROIs) covering brain tissue, the whole tumor (WHT) and the normal appearing white matter on the contralateral side (NAWM) for each patient individually. For each of these ROIs, the total number of voxels included in this mask (# total), the number of excluded voxels (# excl), as well as the percentage of excluded voxels of the total number of voxels (% excl) are listed.

Pat. no.	Brain Mask			Whole Tumor Mask (WHT)			White Matter Mask (NAWM)		
	# total	# excl	% excl	# total	# excl	% excl	# total	# excl	% excl
**1**	4531	668	15	22	0	0	6	0	0
**2**	6286	737	12	152	20	13	2	0	0
**3**	7355	1157	16	422	172	41	9	0	0
**4**	5570	521	9	252	24	10	8	0	0
**5**	5429	1197	22	244	38	16	3	0	0
**6**	5205	950	18	339	60	18	9	0	0
**7**	6351	949	15	121	22	18	6	0	0
**8**	6622	636	10	246	30	12	8	0	0
**9**	7303	460	6	109	14	13	10	0	0
**10**	6502	464	7	201	9	4	7	0	0
**11**	6857	711	10	355	89	25	11	0	0

Table S4.2: Number of excluded voxels for the regions-of-interest (ROIs) covering the gadolinium-contrast enhancement (HGG-CE) and edema (HGG-EDM) for the high grad glioma (HGG), as well as the non-enhancing T_2 hyper intensity (LGG-NCE) for low grade glioma (LGG) for each patient individually. For each of these ROIs, the total number of voxels included in this mask (# total), the number of excluded voxels (# excl), as well as the percentage of excluded voxels of the total number of voxels (% excl) are listed.

Pat. no.	HGG-CE Mask			HGG-EDM Tumor Mask			LGG-NCE Mask		
	# total	# excl	% excl	# total	# excl	% excl	# total	# excl	% excl
**1**	12	0	0	6	0	0			
**2**	64	6	9	77	14	18			
**3**	126	76	60	266	69	26			
**4**	153	21	14	53	0	0			
**5**	70	16	23	244	38	16			
**6**	90	28	31	204	17	8			
**7**	56	10	18	70	14	20			
**8**	173	26	15	13	0	0			
**9**							109	14	13
**10**							201	9	4
**11**	78	39	50	277	50	18			

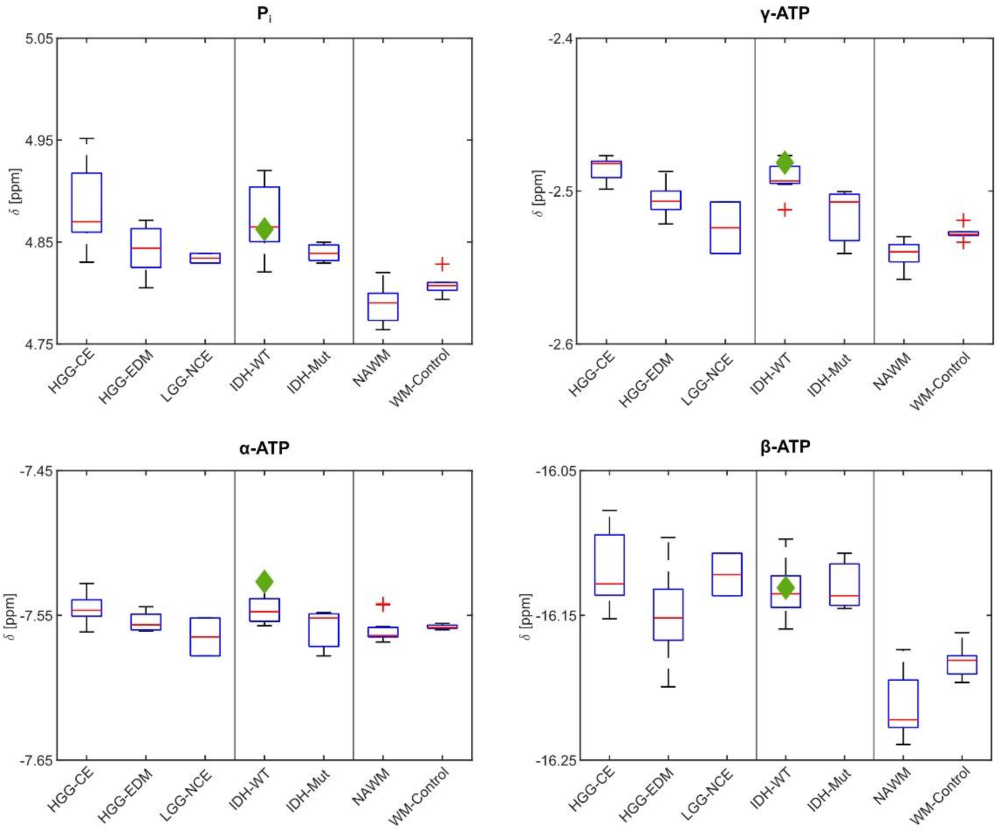

Figure S4: Comparison of mean chemical shifts when no exclusion criterion is applied based on the amplitude CRLBs. Besides that, the figure is analogously to Figure 4 in the main manuscript.

S5 – Statistical Analyses

Despite the small sample sizes in this study, additional statistical analyses were performed in order to complement the solely qualitative comparison of mean chemical shifts between different tumor tissue types. To first test whether the mean chemical shifts of each group are most probably normally distributed, the Shapiro-Wilk test was applied. Table S5.1 lists the W- and p-values for each group and each of the investigated chemical shifts. According to the Shapiro-Wilk test, the values of all groups are normally distributed, except for the chemical shift of α-ATP in the normal-appearing white matter (NAWM) ROI. For the LGG-NCE group, the test could not be applied due to the small sample size ($n = 2$).

Following that analysis, the differences between two groups each were tested for significance in different ways.

For the comparison of chemical shifts to values from normal-appearing white matter (NAWM) combined from all patients ($n = 11$), independent t-tests were used for all cases except for the chemical shift of α-ATP. For α-ATP, the Mann-Whitney U test was applied based on the non-normal distribution according to the Shapiro-Wilk test (cf. Table S5.1). The results of the significance tests and corresponding power analyses are shown in Tables S5.2 and S5.3. For high grade glioma, all chemical shifts are significantly higher in tumor tissue than in NAWM for both the HGG-CE and the HGG-EDM ROI (except for α-ATP, where only the values for HGG-CE are significantly different from NAWM). For low grade glioma, only the chemical shift of P_i is significantly higher than in NAWM. Further, for patients with IDH-WT, the chemical shifts of P_i and ATP in the whole tumor ROI are significantly different from NAWM, whereas for patients with IDH-Mut, only the chemical shifts of P_i and β-ATP differ significantly from NAWM. When comparing values from the NAWM of patients with values from the control group (WM-Control), the chemical shifts of γ- and β-ATP show significant differences with sufficient statistical power.

For the comparison between different tumor sub-compartments for high grade glioma (HGG-CE / HGG-EDM), a paired t-test was performed, as both values are coming from the same patients. For comparing with the values from low grade glioma (HGG-CE / LGG-NCE and HGG-EDM / LGG-CE), the Mann-Whitney U test was used, because values in the LGG-NCE group might not be normally distributed (cf. Table S5.1). For the comparison between different IDH mutation states, an independent t-test was applied. The results of the significance tests and corresponding power analyses are shown in Tables S5.4 and S5.5. According to the performed tests, the difference between HGG-CE and HGG-EDM was significant for the chemical shifts of P_i, γ- and α-ATP. However, according to the power analyses (Table S5.5) only the difference between HGG-CE and HGG-EDM for P_i and γ-ATP is likely to be a true effect.

All tests were also performed accordingly for the parameters pH, Mg and Ion estimated by the look-up algorithm described in section 2.3 in the main text (Tables S5.6-S5.10). When comparing to values from NAWM, the parameters pH and Mg are significantly different (with sufficient statistical power) in the HGG-CE and HGG-EDM ROIs, as well as for the whole tumor ROIs of both IDH-WT and IDH-Mut patients. For the comparison between different tumor tissue types, no significant differences for the parameters pH, Mg and Ion were observed. When comparing value from NAWM with WM-Control, pH and Mg show significant differences but with insufficient statistical power.

Table S5.1: Results of the Shapiro-Wilk Test for the mean chemical shifts of P_i and ATP for each of the investigated groups (i.e. different tumor sub-compartments, different IDH mutation status, normal appearing white matter (NAWM) and white matter from a healthy control group (WM-Control)). For the LGG-NCE group, the test could not be applied (NA) due to the small sample size (n = 2).

**Shapiro-Wilk Test**														
chemical shift	**HGG-CE**		**HGG-EDM**		**LGG-NCE**		**IDH-WT**		**IDH-Mut**		**NAWM**		**WM-Control**	
	W	p	W	p	W	p	W	p	W	p	W	p	W	p
**P_i**	0.89	0.25	0.95	0.71	NA	NA	0.96	0.83	1.00	0.90	0.93	0.44	0.92	0.51
**γ-ATP**	0.91	0.34	0.98	0.94	NA	NA	0.95	0.77	0.84	0.23	0.93	0.43	0.90	0.38
**α-ATP**	0.96	0.83	0.91	0.39	NA	NA	0.95	0.73	0.82	0.17	0.75	0.002	0.95	0.76
**β-ATP**	0.92	0.39	0.93	0.53	NA	NA	0.94	0.67	0.96	0.59	0.89	0.13	0.95	0.74

Table S5.2: Results of the significance tests applied for comparing the mean chemical shifts of P_i and ATP in the different tumor tissue ROIs with the values from the normal-appearing white matter (NAWM) ROI. Additionally, the last column shows the results for comparing the NAWM values from the patients with values from white matter from a healthy control group (WM-Control). For all comparisons, an independent t-test was applied except for the case of α-ATP, for which a Mann-Whitney U test was used. p-values < 0.05 are highlighted in orange.

**Significance tests**						
p-values						
chemical shift	**HGG-CE / NAWM**	**HGG-EDM / NAWM**	**LGG-NCE / NAWM**	**IDH-WT / NAWM**	**IDH-Mut / NAWM**	**NAWM / WM-Control**
**P_i**	6.9E-05	1.6E-04	6.7E-04	8.0E-04	3.1E-04	1.2E-02
**γ-ATP**	7.2E-11	7.3E-06	4.5E-01	1.5E-06	1.5E-01	8.3E-04
**α-ATP**	3.6E-03	5.1E-02	7.7E-01	1.1E-02	8.8E-01	9.8E-02
**β-ATP**	4.5E-08	5.8E-04	6.1E-02	1.9E-05	9.2E-03	1.4E-03

Table S5.3: Results of the power analyses performed corresponding to the tests in Table S5.2. Cases with a power ≥ 0.8 are highlighted in green.

**Power Analyses**						
p-values						
chemical shift	**HGG-CE / NAWM**	**HGG-EDM / NAWM**	**LGG-NCE / NAWM**	**IDH-WT / NAWM**	**IDH-Mut / NAWM**	**NAWM / WM-Control**
**P_i**	1.00	1.00	0.95	1.00	1.00	0.71
**γ-ATP**	1.00	1.00	0.09	1.00	0.33	0.94
**α-ATP**	0.95	0.31	0.05	0.88	0.05	0.08
**β-ATP**	1.00	0.99	0.56	1.00	0.96	0.92

Table S5.4: Results of the significance tests applied for comparing the mean chemical shifts of P_i and ATP between two different tumor tissues and IDH mutation status each. For the comparison

between HGG-CE and HGG-EDM, a paired t-test was performed. For the comparison between HGG-CE / LGG-NCE and HGG-EDM / LGG-NCE the Mann-Whitney U test was used, because values for LGG-NCE might not be normally distributed (cf. Table S5.1). For the comparison between different IDH mutation status, an independent t-test was applied. p-values < 0.05 are highlighted in orange.

**Significance tests**				
p-values				
chemical shift	**HGG-CE / HGG-EDM**	**HGG-CE / LGG-NCE**	**HGG-EDM / LGG-NCE**	**IDH-WT / IDH-mut**
**P_i**	0.014	0.178	0.533	0.098
**γ-ATP**	0.004	0.044	0.400	0.174
**α-ATP**	0.040	0.089	0.533	0.257
**β-ATP**	0.065	0.711	0.267	0.471

Table S5.5: Results of the power analyses performed corresponding to the tests in Table S5.4. Cases with a power ≥ 0.8 are highlighted in green.

**Power Analyses**				
p-values				
chemical shift	**HGG-CE / HGG-EDM**	**HGG-CE / LGG-NCE**	**HGG-EDM / LGG-NCE**	**IDH-WT / IDH-mut**
**P_i**	0.80	0.51	0.06	0.26
**γ-ATP**	0.95	0.19	0.09	0.27
**α-ATP**	0.58	0.14	0.07	0.19
**β-ATP**	0.47	0.06	0.18	0.10

Table S5.6: Results of the Shapiro-Wilk Test for the parameters pH, magnesium ion content (Mg) and Ion (being a relative surrogate measure for the ionic strength) for each of the investigated groups (i.e. different tumor sub-compartments, different IDH mutation status, normal appearing white matter (NAWM) and white matter from a healthy control group (WM-Control)). For the LGG-NCE group, the test could not be applied (NA) due to the small sample size (n = 2).

**Shapiro-Wilk Test**														
parameter	**HGG-CE**		**HGG-EDM**		**LGG-NCE**		**IDH-WT**		**IDH-Mut**		**NAWM**		**WM-Control**	
	W	p	W	p	W	p	W	p	W	p	W	p	W	p
**pH**	0.86	0.11	0.99	0.99	NA	NA	0.94	0.67	0.78	0.06	0.93	0.38	0.96	0.85
**Mg**	0.91	0.37	0.90	0.31	NA	NA	0.90	0.35	0.99	0.79	0.91	0.26	0.87	0.24
**Ion**	0.93	0.51	0.94	0.59	NA	NA	0.93	0.55	0.95	0.55	0.95	0.69	0.94	0.65

Table S5.7: Results of the significance tests applied for comparing the parameter pH, Mg and Ion in the different ROIs with the values from the normal-appearing white matter (NAWM) ROI. The last column shows the results for comparing the NAWM values from the patients with values from white

matter from a healthy control group (WM-Control). For all comparisons, an independent t-test was used. p-values < 0.05 are highlighted in orange.

Significance tests						
p-values						
parameter	**HGG-CE / NAWM**	**HGG-EDM / NAWM**	**LGG-NCE / NAWM**	**IDH-WT / NAWM**	**IDH-Mut / NAWM**	**NAWM / WM-Control**
**pH**	7.5E-04	1.3E-03	1.1E-01	2.4E-03	1.1E-02	9.0E-03
**Mg**	1.8E-03	1.7E-04	8.1E-03	1.5E-03	1.9E-04	2.5E-02
**Ion**	7.2E-01	5.9E-01	2.8E-01	6.3E-01	2.5E-01	3.1E-01

Table S5.8: Results of the power analyses performed corresponding to the tests in Table S5.7. Cases with a power > 0.8 are highlighted in green.

Power Analyses						
p-values						
parameter	**HGG-CE / NAWM**	**HGG-EDM / NAWM**	**LGG-NCE / NAWM**	**IDH-WT / NAWM**	**IDH-Mut / NAWM**	**NAWM / WM-Control**
**pH**	0.99	0.96	0.35	0.97	0.89	0.69
**Mg**	0.96	0.99	0.68	0.96	0.93	0.54
**Ion**	0.06	0.08	0.15	0.07	0.20	0.15

Table S5.9: Results of the significance tests applied for comparing the parameter pH, Mg and Ion between two groups each. For the comparison between HGG-CE and HGG-EDM, a paired t-test was performed. For the comparison between HGG-CE / LGG-NCE and HGG-EDM / LGG-NCE the Mann-Whitney U test was used, because values for LGG-NCE might not be normally distributed (cf. Table S5.6). For the comparison between different IDH mutation status, an independent t-test was applied.

Significance tests				
p-values				
parameter	**HGG-CE / HGG-EDM**	**HGG-CE / LGG-NCE**	**HGG-EDM / LGG-NCE**	**IDH-WT / IDH-mut**
**pH**	0.11	0.71	0.27	0.83
**Mg**	0.58	0.89	0.53	0.66
**Ion**	0.95	0.09	0.09	0.27

Table S5.10: Results of the power analyses performed corresponding to the tests in Table S5.9.

Power Analyses				
p-values				
parameter	**HGG-CE / HGG-EDM**	**HGG-CE / LGG-NCE**	**HGG-EDM / LGG-NCE**	**IDH-WT / IDH-mut**
**pH**	0.35	0.05	0.09	0.05
**Mg**	0.08	0.11	0.07	0.06
**Ion**	0.05	0.15	0.15	0.18

S6 – Mean chemical shifts referenced to α-ATP

As an alternative to referencing the chemical shifts to phosphocreatine (PCr), a common alternative is to reference to α-ATP. In the following Figure S6, we are showing the mean chemical shifts of P_i, γ- and β-ATP all referenced to the chemical shift of α-ATP. The differences of the mean chemical shifts show the same pattern as when referenced to PCr (cf. Figure 4 of the main manuscript).

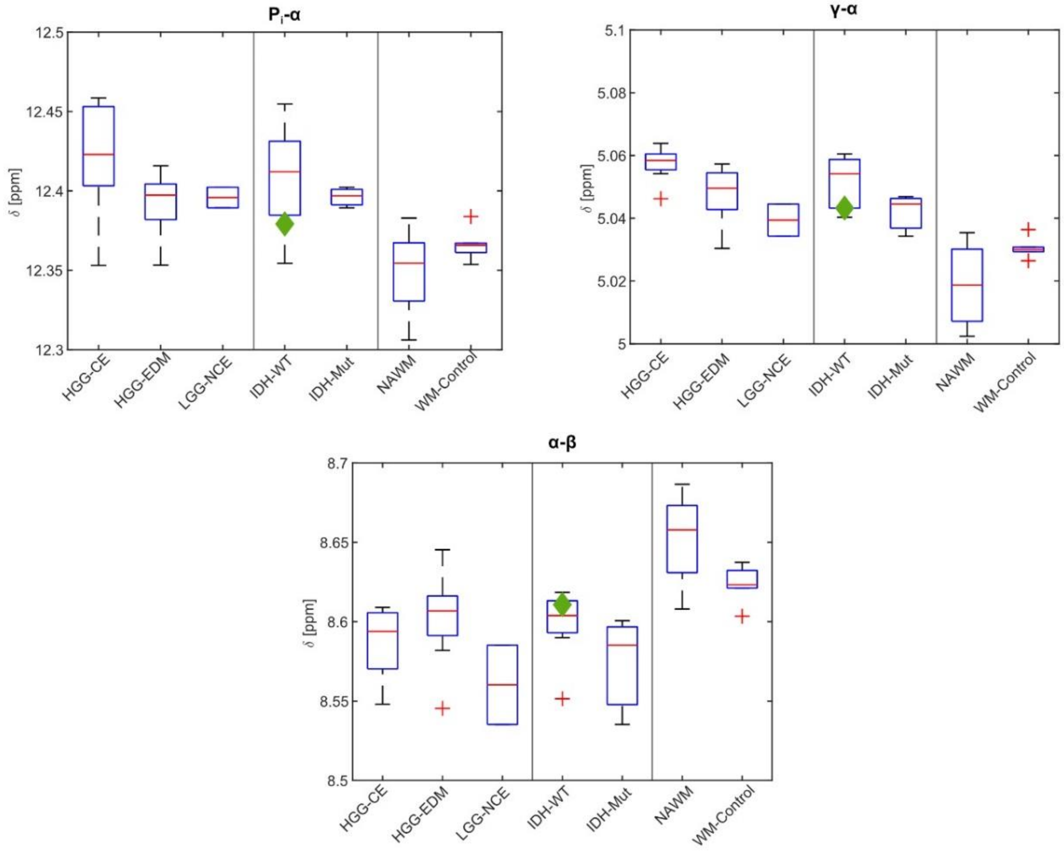

Figure S6: Comparison of the mean chemical shifts of P_i, γ- and β-ATP all referenced to the chemical shift of α-ATP averaged across different regions-of-interest (ROIs) and different groups of patients. In the left sections, different tumor sub-compartments are compared: For patients with high-grade glioma (HGG, n = 8), the mean chemical shifts across the contrast enhancing areas (HGG-CE) and the edema (HGG-EDM) are shown, and for the patients with low-grade glioma (LGG, n = 2) the mean chemical shifts across the respective non-contrast enhanced areas (LGG-NCE). In the central sections, the mean chemical shifts across the respective whole tumor volume are compared between patients with different IDH-mutation status: IDH-wildtype (WT) (n = 7) and IDH-mutant (mut) (n = 3). The data point belonging to the dataset from the patient with pleomorphic glioma is marked a green diamond. In the right sections of each plot, the mean chemical shifts across the normal-appearing white matter ROI (NAWM) from all 11 patients, as well as values from healthy white matter of a control group (WM-control) are shown (here also the patient with pleomorphic glioma is included). The red bar of the boxplots depicts the median across the respective groups of patients.

--- References ---

1. Paech D, Weckesser N, Franke VL, et al. Whole-Brain Intracellular pH Mapping of Gliomas Using High-Resolution 31P MR Spectroscopic Imaging at 7.0 T. Radiol. Imaging Cancer 2024;6 doi: 10.1148/rycan.220127.

2. Louis DN, Perry A, Reifenberger · Guido, et al. The 2016 World Health Organization Classification of Tumors of the Central Nervous System: a summary. Acta Neuropathol. 2016;131:803–820 doi: 10.1007/s00401-016-1545-1

3. Lin A, Andronesi O, Bogner W, et al. Minimum Reporting Standards for in vivo Magnetic Resonance Spectroscopy (MRSinMRS): Experts' consensus recommendations. NMR Biomed. 2021;34:e4484 doi: 10.1002/NBM.4484.

4. Vanhamme L, Van Huffel S. AMARES: Advanced Method for Accurate, Robust and Efficient Spectral fitting of MRS data with use of prior knowledge. J. Magn. Reson. 1997;43:1–2.
